# Genetic diversity analysis and population structure of some Iranian Fenugreek (*Trigonella foenum-graecum* L.) landraces using SRAP Markers

**Published:** 2019-12

**Authors:** Marzieh Amiriyan, Abdolali Shojaeiyan, Abbas Yadollahi, Masoud Maleki, Zeinab Bahari

**Affiliations:** Department of Horticultural Science, Faculty of Agriculture, Tarbiat Modares University, Tehran, Iran

**Keywords:** PCOA, GenAlex, Dendrogram, Pearson correlation, Genetic Diversity, Mantel test

## Abstract

Fenugreek is one of the important edible and medicinal vegetables that have a long history of cultivation and consumption. Characterize the extent of the genetic diversity among landraces will provide a good context for future breeding programs and genetic resource preservation. Genetic diversity and population structure of 88 individuals of eight landraces of Iranian fenugreek evaluated based on SRAP markers. Seventy-two bands generated from 6 primers in which 56 (80.11%) band were polymorph. Hamadan landrace showed the lowest values of percentage of polymorphic loci (67.86), Nei's gene diversity index (0.24), number of effective alleles (1.40) and Shannon’s Information index (0.36). Nei’s genetic distance matrix revealed the highest genetic distance between Hamadan and Yazd (0.203) and the highest genetic similarity between Mahallat and Varamin (0.036) landraces. The most gene flow was between Mahallat and Varamin landraces (Nm=8.36) and the least was between Shiraz and Hamadan landraces (Nm=0.66). An extent admixture of alleles between the Iranian fenugreek landraces was observed by the population structure. Mantel test indicated that the genetic differentiation and gene flow is not associated with geographic distance in Iranian fenugreek landraces. Our observations indicated SRAP is an efficient technique to reveal genetic diversity and population structure of Iranian fenugreek landrace.

## INTRODUCTION

Fenugreek (*Trigonella foenum-graecum* L.) is an annual, herbaceous, dicotyledonous, diploid, self-pollinated plant which belongs to the subfamily Papilionaceae in Fabaceae family [1]. Many species of this family are known to have high nutritional value and consumed in the forms of pasture seeds, oil seeds or dried nuts, fiber and resin [2]. Fenugreek is one of the oldest medicinal plants which its leaves are used as the vegetable and are a rich source of calcium, iron, carotene, and vitamins A and D [3, 4]. It has a strong seed mucilage as well as laxative, carminative, soothing, diuretic, mucus-inductive, restorative, and parasiticidal effects; while its impact on the treatment of ulcers in the mouth, dried or cracked lips, and inflammation of the intestines and duodenum are well-known as well [5]. A wide range of health benefits is associated with the consumption of fenugreek, including; anti-cancer, anti-tumor, anti-diabetic, anti-inflammatory, antipyretic, and antioxidant effects; positive impact on breast milk increase and cholesterol and blood pressure reduction. Cases of use as a heart tonic have been reported by some studies [6-9].

Widely, different opinions are proposed for the likely origin of *T. foenum-graecum*. Some authors have suggested that fenugreek is native to the Mediterranean region, Asian regions, such as Punjab and Kashmir, deserts of Mesopotamia and Persia, Asia Minor, and Southern Europe, such as Greece, Italy, Turkey and Spain [8, 10]. Nevertheless, the origin of fenugreek is more known to be Asia rather than Southern Europe [5, 8]. Nowadays, this plant is widely grown all over the world, especially in India (especially Rajasthan state), Egypt, Ethiopia, and the United Kingdom, due to high adaptability of fenugreek to different climatic and growth conditions [11-13]. However, significant *Trigonella* producing areas extend from Iran to Northern India [14].

Some studies investigated the genetic diversity of fenugreek by various genetic and morphological markers, such as random amplification of polymorphic DNA (RAPD), simple sequence repeat (SSR), inter sequence simple repeat (ISSR) and amplified fragment length polymorphism (AFLP) markers [10, 11, 14-17]. 

SRAP technique is a polymerase chain reaction (PCR) based marker system first developed by Li and Quiros in 2001 to exclusive amplification of genome-encoding regions, mapping and gene tagging in *Brassica*. SRAPs have been widely employed for the construction of linkage maps, identification of quantitative trait loci (QTL), analyses of inter- and intraspecific systematic of landraces, hybridization, biogeography, and conservation genetics. [18, 19]. Up to now, SRAP markers have been applied to scrutinize genetic diversity in miscellaneous crops, herbs, and trees such as shiitake mushroom (*Lentinula edodes*) [20], bermudagrass (*Cynodon dactylon* (L.) Pers.) [21], watermelon (*Citrullus lanatus*) [22], pistachio (*Pistacia vera* L.) [23], purslane (*Portulaca* L.) [24], and black cumin (*Nigella* *sativa* L.) [25]. Nonetheless, the genetic diversity of fenugreek populations has not yet been examined by SRAP markers.

The main goals of this study were to estimate the extent of polymorphism in the Iranian fenugreek landraces using SRAP primers, evaluate the effectiveness of SRAP in distinguishing the interspecific diversity of fenugreek and laying the foundations for purposive breeding programs to take full advantages of desirable genotypes.

## MATERIALS AND METHODS

**Plant Material and DNA Extraction: **Seeds of eight landraces of fenugreek collected from different regions of Iran (Table 1) and grew in a greenhouse provided by the department of horticultural science at Tarbiat Modares University. Then, the true leaves of 11 individuals of each landrace were harvested and were put in sealed bags containing silica gel to accelerate the drying process. Afterward, 10 mg of dried leaves of each individual were pulverized by the Mixer Mill Machine (MM 400; Retsch, Germany), followed by extraction of the DNA of samples with the modified CTAB method [26]. 

**Table 1:** Geographical location of Iranian fenugreek landraces collected for the present study

**Collection sites**			**Geographical location**		
**Cities**	**Abbrviation**	**Provinces**	**Region**	**Longitude (E)**	**Latitude (N)**
**Shiraz**	SHZ	Fars	Southern Iran	29'30	55'00
**Sanandij**	SAN	Kurdistan	Western Iran	36'00	47'00
**Kerman**	KER	Kerman	Southern Iran	30'15	57'01
**Mahallat**	MAH	Markazi	Central Iran	33'55	50'30
**Mashhad**	MAS	Khorasan Razavi	Eastern Iran	36'20	59'35
**Hamadan**	HAM	Hamadan	Western Iran	35'00	49'00
**Varamin**	VAR	Tehran	Central Iran	35'44	51'30
**Yazd**	YAZ	Yazd	Southern Iran	32'00	55'00

**DNA amplification: **The polymerase chain reaction (PCR) reaction mixture in 10 μl volume contained: 2.46 μl of sterile distilled water, 1 μl of PCR buffer, 1.5 μL of MgCl_2 _(15 mM), 0.8 μL of dNTP, 0.33 μL of forward primer (10 ρmol μL^-1^), 0.33 μL of reverse primer (10 ρmol μL^-1^), 0.08 μL of Taq DNA polymerase (5 unit, Smart taq DNA polymerase, Sinaclon, Iran), and 3.5 μL of DNA (20 ng). SRAP PCR reactions were carried out using a Thermocycler Machine (BIORAD, C 1000™) with an initial step at 95˚C for 3 min, followed by 5 cycles of 1 min at 94˚C, 1 min at 35˚C, and 1 min at 72˚C, then, 35 cycles of 1 min at 94˚C, 1 min at 35˚C, and 1 min at 72˚C, and a final extension of 3 min at 72°C. PCR products were analyzed on 10% non-denaturing polyacrylamide and visualized by silver staining [27]. 

**Data Analysis** **: **Clear and reproducible fragments were scored in a binary matrix with a present (1) or absent (0) approach for each sample and used for the following analysis. Nei’s genetic distance was used to draw dendrogram for the landraces based on the Neighbor-Joining clustering algorithm using Powermarker Ver.3.25 (Fig. 2) [28]. The dendrogram was visualized using Fig Tree v1.4.4 software [29] .

Polymorphic information content (PIC) of SRAP primers was calculated in Excel using the formula; PICi=2 f_i_ (1- f_i_), where the PICi is the polymorphic information content of marker ‘i’, *f*_i_ is the frequency of the amplified fragments (band present) and 1–f_i_ is the frequency of non-amplified fragments (band absent), range from 0 to 0.5 for dominant makers [30, 31]. GenAlEx 6.501 software [32] was used to calculate the statistical measures of genetic variation (i.e., Nei’s genetic distance [33], Shannon’s information index (I), percentage of polymorphic loci (PPL), number of different alleles (N_a_), number of effective alleles (N_e_) and Principal coordinate analysis (PCoA)(. Partitioning of variance within and between landraces was assessed using Analysis of molecular variance (AMOVA). PhiPT (analog of F-statistic) was applied to evaluate Pairwise measures of gene flow and differentiation between landraces. Both AMOVA and PhiPT were performed in GenAlEx 6.501 with 999 permutations [32]. The number of migrants per generation (Nm) was estimated using the following formula in Excel file [34-36]; 

$\mathrm{Nm}=0.25\times [\left(\frac{1}{\mathrm{PHIPT}}\right)-1]$

Mantel tests were performed in GenAlEx with 9999 permutations between pairwise Nm values and geographic distance (km) for all landraces: STRUCTURE software ver. 2.3.4 [37], which is a model-based Bayesian method, used to determine the structure of the landraces. For each analysis, we set the number of genetically distinct groups (K) from 2 to 7 (with 10 iterations for each K) and ran the program with 10,000-initial burn-in followed by 100,000 Markov Chain Monte Carlo (MCMC) replications. Afterward, the result of the STRUCTURE run was uploaded to Structure Harvester website program [38] to obtain number of K groups that best fit the data. 

## RESULTS

The six primer combinations amplified a total of 72 alleles, of which 56 polymorphic bands were detected (Tables 2 and 3). The percent of total polymorphic bands produced by each primer combinations ranged from 41.6% (for Me1-Em4) to 100% (for Me3-Em3), with an average of 80.11% (Table 4). The polymorphic information content (PIC) is represented as the ability of each primer combinations to differentiate the landraces. The maximum and minimum values of PIC was observed in Me1-Em4 (0.46) and for Me3-Em3 (0.31), respectively. The average PIC was 0.36 (Table 4).

Several genetic diversity measures were calculated such as the PPL, N_a_, N_e_, Nei's gene diversity index (*h*) and (I) (Table 4). The averages of N_e_, N_a_, *h* and I were 1.507, 1.569, 0.286 and 0.421, respectively. The average PPL per landrace was 76.34. The highest (83.93%) and lowest (67.86%) values of PPL were observed in Kerman and Hamadan landraces, respectively. Values of the Shannon's information index (I) ranged from 0.36 (Hamadan) to 0.45 (Mashhad), indicating a moderate allelic frequency and uneven distribution. The highest (0.35) and lowest (0.24) values of *h* were observed in Yazd and Hamadan landraces, respectively. The value of N_a_ varied from 1.45 (Varamin) to 1.68 (Kerman), and the N_e_ ranged from 1.40 (Hamadan) to 1.65 (Yazd) (Table 4), which indicates the presence of high genetic diversity among the landraces. Hamadan landrace had the lowest values for N_e_, I, *h*, and PPL indices demonstrate that the most homogeneity among the landraces can be found in Hamadan landrace (Table 4). 

**Table 2:** Characteristics of SRAP primers in evaluation of genetic diversity of Iranian fenugreek landraces

**No.**	**Primer Sequences (5′-3′)**	**Primer name**	**Direction **
1	TGAGTCCAAACCGGATA	Me1	Forward
2	TGAGTCCAAACCGGAGC	Me2	
3	TGAGTCCAAACCGGAAT	Me3	
4	TGAGTCCAAACCGGACC	Me4	
5	TGAGTCCAAACCGGAAG	Me5	
6	GACTGCGTACGAATTAAT	Em1	Reverse
7	GACTGCGTACGAATTTGC	Em2	
8	GACTGCGTACGAATTGAC	Em3	
9	GACTGCGTACGAATTTGA	Em4	
10	GACTGCGTACGAATTAAC	Em5	

**Table 3:** Polymorphism information among Iranian fenugreek landraces, using SRAP primers

**NO**	**Primer combinations**	**Perc. of polymorphic bands**	**No. of polymorphic bands**	**No. of** **Scored bands**	**PIC-Value**
1	Me1- Em4	41.6	5	12	0.46
2	Me3- Em3	100	10	10	0.31
3	Me3- Em5	81.25	13	16	0.34
4	Me4- Em3	85.71	6	7	0.38
5	Me4- Em4	83.3	15	18	0.36
6	Me5- Em3	88.8	8	9	0.32
	Average	80.11	9.5	12	0.36

**Table 4:** Genetic diversity measures for Iranian fenugreek landraces

**NO.**	**Landraces **	**N**_a_ ** ± sd**	**N**_e_ ** ± sd**	**I** ** ± sd**	***h*** ** ± sd**	**PPL** ** %**
1	Shiraz	1.55 ± 0.11	1.50 ± 0.05	0.43 ± 0.03	0.29 ± 0.02	76.79
2	Sanandij	1.64 ± 0.10	1.50 ± 0.05	0.43 ± 0.03	0.29 ± 0.02	78.57
3	Kerman	1.68 ± 0.10	1.49 ± 0.04	0.44 ± 0.03	0.29 ± 0.02	83.93
4	Mahallat	1.52 ± 0.11	1.46 ± 0.05	0.40 ± 0.04	0.27 ± 0.02	73.21
5	Mashhad	1.61 ± 0.10	1.54 ± 0.05	0.45 ± 0.03	0.31 ± 0.02	80.36
6	Hamadan	1.50 ± 0.10	1.40 ± 0.05	0.36 ± 0.40	0.24 ± 0.02	67.86
7	Varamin	1.45 ± 0.12	1.44 ± 0.05	0.38 ± 0.04	0.25 ± 0.03	69.64
8	Yazd	1.61 ± 0.11	1.65 ± 0.05	0.50 ± 0.03	0.35 ± 0.02	80.36
	Mean	1.57 ± 0.04	1.50 ± 0.02	0.42 ± 0.01	0.28 ± 0.01	76.34

sd; standard deviation, N_a_; number of different alleles, N_e_; number of effective alleles, I; Shannon's information index, *h*; Nei's gene diversity index, PPL; percentage of polymorphic loci

According to the Pearson correlation analysis there was a positive significant relationship between diversity measures (Table 5), that was in accordance with the correlation analysis on data that were derived from previous studies on other crops [30, 39-40].

AMOVA indicated that most significant genetic diversity (86%) is ascribed to within landraces variation rather than among the landraces (14%), with a value of 0.14 for PhiPT (P<0.001) (Table 6). The genetic distance between landraces ranged from 0.048 to 0.222. The highest genetic distance was observed between Hamadan and Yazd landraces and the lowest distance-or, in other words, the highest genetic similarity-was between the Mahallat and Varamin landraces. In the present study, the most gene flow was between Mahallat and Varamin landraces (Nm=8.36) and the least was between Shiraz and Hamadan landraces (Nm=0.66) (Table 7).

**Table 5:** Pearson correlation analysis of Genetic diversity measures for Iranian fenugreek landraces

	**N** ***a***	**N** ***e***	**I**	***h***
N*e*	0.556			
I	0.718*	0.971**		
h	0.663	0.987**	0.992**	
PPL	0.932**	0.698*	0.848**	0.789*

N*a*; number of different alleles, N*e*; number of effective alleles, I; Shannon's information index, *h*; Nei's gene diversity index, PPL; percentage of polymorphic loci

**Table 6:** Analysis of molecular variance using SRAP molecular markers in fenugreek landraces

**Source of Variation**	**df**	**MS**	**Est. Var. **	% *	**Nm**	***P *** **value**
Among landraces	7	24.911	1.46	14	1.51	0.001
Within landraces	80	8.84	8.84	86		
Total	87		10.30	100		

df: degree of freedom; MS: mean of squares, Est. Var.: estimated variation . *percent of total variation

**Table 7:** Gene flow (Nm, above diagonal) and Nei’s genetic distance (below diagonal) of the fenugreek landraces

**Landraces**	**SHZ**	**SAN**	**KER**	**MAH**	**MAS**	**HAM**	**VAR**	**YAZ**
Shiraz	0	1.03	1.18	1.58	2.10	0.66	1.37	3.37
Sanandij	0.162	0.00	2.18	1.83	1.12	0.93	2.07	1.04
Kerman	0.148	0.097	0.00	5.14	2.18	1.14	2.98	1.65
Mahallat	0.113	0.102	0.062	0.00	2.38	0.87	8.36	1.75
Mashhad	0.104	0.159	0.102	0.091	0.00	2.41	2.33	2.22
Hamadan	0.205	0.152	0.131	0.153	0.083	0.00	1.34	0.73
Varamin	0.120	0.091	0.075	0.048	0.089	0.106	0.00	1.69
Yazd	0.089	0.188	0.134	0.122	0.117	0.222	0.119	0.00

**Figure 1:**
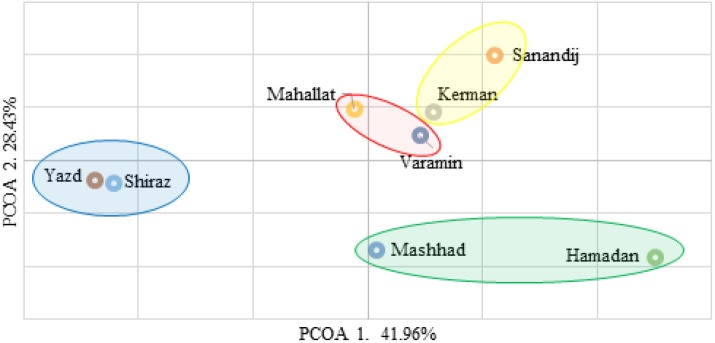
Two-dimensional plot from Principal Coordinate Analysis of genetic distance matrix of Iranian fenugreek landraces

The principal coordinate analysis was used to further assess genetic relationships among fenugreek landraces. The two-dimensional scatter plot has indicated that the first two PCoA axes accounted for 41.96% and 28.43% of the genetic variation, respectively (Fig. 1). This analysis separated the fenugreek landraces into four groups that are indicated in figure 1.

**Figure 2:**
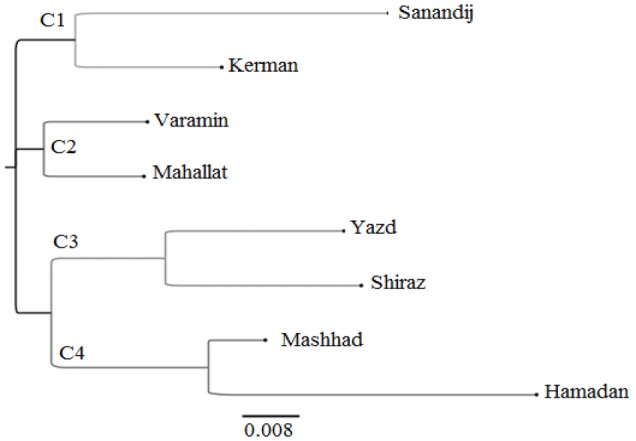
Neighbor-Joining dendrogram of SRAP data of Iranian fenugreek landraces

A dendrogram was generated based on Nei’s genetic distance matrix of the SRAP data using the Neighbor-joining algorithm (Fig. 2). Based on the dendrogram, Iranian fenugreek landraces were clustered into four clusters. The clustering results according to genetic distance were consistent with the results from PCoA analysis. Each cluster contained two landraces. Cluster 1 (C1) contained Sanandij and Kerman landraces. Cluster 2 (C2) contained Varamin and Mahallat landraces. Cluster 3 (C3) cluster included Yazd and Shiraz landraces. Mashhad and Hamadan landraces placed in Cluster 4 (C4). 

**Figure 3:**
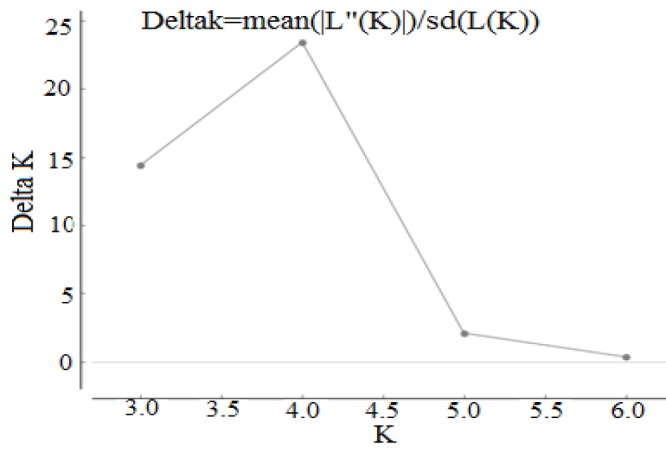
Inference of optimum K based on delta K for SRAP data of 8 Iranian fenugreek landraces

**Figure 4:**
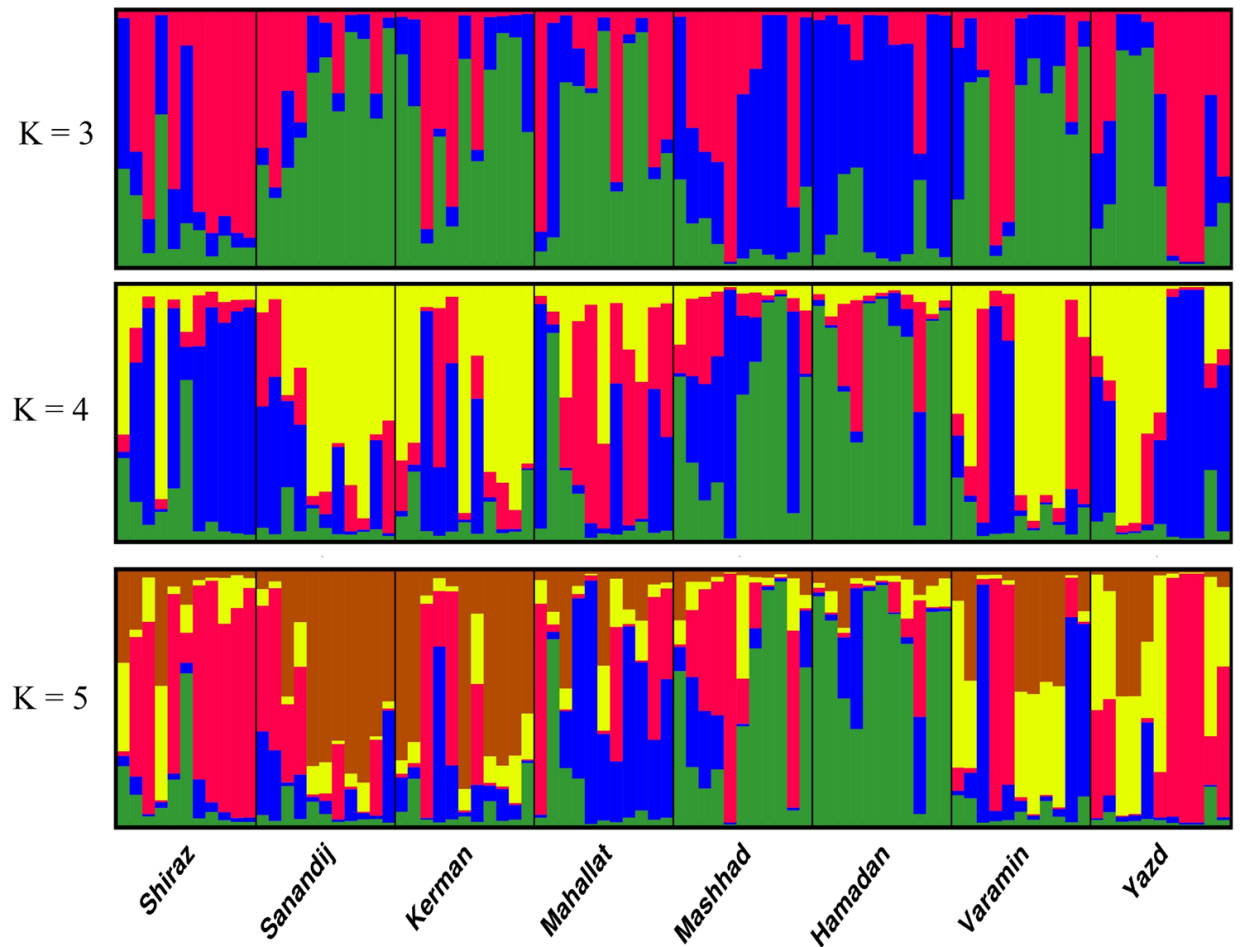
Structure plot of fenugreek landraces based on SRAP data. Each landrace is represented by a single vertical bar, which is partitioned into different colors. Each color represents a genetic cluster and the colored segments shows the individual’s estimated ancestry proportion to each of the genetic clusters.

The structure results of K=3 to K=5 are shown in Figure 4. Maximum *ΔK *was found at *K*=4 (Fig. 3), and this was considered as an optimum number of population for Iranian fenugreek landraces. The STRUCTURE analysis results were consistent with the pattern of diversity revealed by the NJ based trees and PCoA analyses of the fenugreek landraces, which were not separated into different groups based on their geographical distance. STRUCTURE plot indicated close genetic relation between Yazd and Shiraz landraces (contain mostly blue colored segments), Mashhad and Hamadan landraces (contain mostly green colored segments), and Sanandij and Kerman landraces (contain mostly yellow colored segments) (Fig. 4). The results of K=3 was similar to K=4, except that at K=3 two cluster (C1 and C2) had been merged, so Sanandij, Kerman, Varamin, and Mahallat landraces placed in a common population. Based on the result of K=5, five populations could be assigned for all the landraces. Based on K=5, the fifth population can be generated by the separation of Mahallat landrace (contain mostly blue colored segments) from Varamin landrace, which already were placed together in the same group at K=4. Based on the structure plot, there were not any salient appearance of similarity between Mahallat and Varamin landraces (Fig. 4), in spite of low genetic distance (high similarity values) between them (Table 7). 

## DISCUSSION

Substantially, fenugreek is a self-pollinated crop and it is expected to observe low genetic diversity within landraces, however, in the present study, a high value of genetic diversity within the studied landraces was observed. Self-pollination intended to diminish genetic diversity in a landrace but many factors can affect this phenomenon, like migration, cross-pollination, and etc. Moreover, the existence of high genetic diversity among and within the studied genotypes proposes Iran as one of the centers of origin or diversity of this crop. Sadeghzade Ahari et al., [15] also reported high genetic diversity in 20 landraces of fenugreek in Iran using RAPD and AFLP markers. 

The average percent of polymorphic bands produced by each primer combination was 80.11% (Table 4). Percentage polymorphism obtained by SRAP markers in our study was significantly higher than Sindhu et al., [14] which reported 55.60% and 50.16% for RAPD and SSR markers, respectively. 

The AMOVA showed a high proportion of the variability was due to within-landraces diversity. This result was confirmed with previous studies on the genetic diversity of fenugreek with different origins [11, 15]. The number of migrants per generation (migration rate) is difficult to measure by direct tracking of individuals, pollen, etc. Furthermore, immigrants may not breed in their new habitat. Subsequently, gene flow instead of the migration rate could be estimated [36]. Gene flow has an important role in the dispersion and differentiation of plant populations. Mainly, in seed plants, gene flow is occurred by seeds or pollen contain foreign genes between groups [41]. Populations with migration rates of more than one migrant per generation (Nm=2 and 4) exhibit no differentiation, while those with less than one migrant per generation (Nm=1/2 and 1/4) differentiate to such an extent that some populations are fixed for alternative alleles [36]. The average of gene flow among the Iranian fenugreek landraces was 1.51, it means the migrant rate is more than one immigrant per generation so that differentiation has not occurred. Besides, gene flow is reversely correlated with the Nei’s genetic distance, as evident in Table 7. Fan et al., [41] reported the high average of gene flow among the *V. ficifolia* populations. Also, according to the Mantel test, genetic differentiation and gene flow were not associated with geographic distance in Iranian fenugreek landraces. Hence, gene flow among studied landraces did not restraint and stop by geographic distances and may be affected by other factors like human effect. Previous genetic diversity investigations by other molecular markers on fenugreek also obtained similar results [10, 14, 17]. Sindhu et al., [14] reported that the genotypes from the same collection region genetically were placed into different clusters and less significant association was observed between genetic and geographical distances.

The population structure of the Iranian fenugreek landraces displayed a very high admixture of alleles and none of them identified as a pure line, which could be connected to cases such as gene flow, cross-pollination among the landraces, and ancestral common genetic content [42]. 

In conclusion, the application of morphological, biochemical, physiological markers, and other molecular marker techniques on more landraces from growing regions of Iran, highly recommended to fully characterize the extent of the genetic diversity among landraces which provides a good context for future breeding programs and genetic resources preservation.

The present study is the first report of using the SRAP markers for evaluating genetic diversity and population genetic structure in fenugreek landraces. In light of the obtained results, it can be said that SRAP is an effective technique in revealing allelic differences and may, therefore, be used in future studies on the genetic diversity of fenugreek crop.
